# The Value of Knowledge and Other Epistemic Standings: A Case for Epistemic Pluralism

**DOI:** 10.1007/s11406-023-00647-8

**Published:** 2023-04-01

**Authors:** Guido Melchior

**Affiliations:** Department of Philosophy, University of Graz, Heinrichstrasse 26/5, Graz, 8010 Austria

**Keywords:** Value of knowledge, Meno problem, Epistemic value, Epistemic pluralism, Knowledge, Theory of knowledge

## Abstract

In epistemology, the concept of knowledge is of distinctive interest. This fact is also reflected in the discussion of epistemic value, which focuses to a large extend on the value problem of knowledge. This discussion suggests that knowledge has an outstanding value among epistemic standings because its value exceeds the value of its constitutive parts. I will argue that the value of knowledge is not outstanding by presenting epistemic standings of checking, transferring knowledge, and proving in court, whose values exceed the value of knowledge in certain contexts. Moreover, the values of these other epistemic standings do not always rely on the value of knowledge. In terms of value, knowledge is not an outstanding epistemic concept. Hence, in terms of value we cannot find support for the privileged position that knowledge enjoys in epistemology.

## The Epistemological Focus on Knowledge

To begin with, let me sketch a broader picture of the context in which this paper is situated. Knowledge is taken to be the central concept of epistemology. For example, introductions into epistemology are often labeled as ‘theory of knowledge’. Other epistemic concepts such as warrant or undefeated justification are often only discussed for the purpose of analyzing knowledge and not as concepts of interest in their own rights.^1^ This focus on knowledge in epistemology does not do justice to the plurality of the epistemic concepts we use in everyday life, including checking, proving, understanding, explaining, arguing, convincing, or discriminating, as many of these concepts are neglected by orthodox epistemology.^2^ This is a shortcoming for at least two reasons. First, these phenomena are interesting in their own rights and deserve our attention. Second, a deeper understanding of these phenomena can lead to a better understanding of the concept knowledge and of puzzles about knowledge in turn. Accordingly, I do not think that the privileged status that knowledge has in contemporary epistemology is justified. Rather, knowledge should be understood as one epistemic concept amongst others to be analyzed. In this sense, I favor conceptual pluralism in epistemology.

The notion of epistemic pluralism is used in different ways. Coliva and Pederson (2017, 2) distinguish several distinct versions of epistemic pluralism as the views (a) that there are several ways of being epistemically justified, (b) that there are several ways of being epistemically warranted, (c) that there are several ways of being epistemically rational, (d) that there are several epistemic desiderata, (e) that there are several epistemic principles, (f) that there are several epistemic methods, and (g) that there are several epistemic goods. Zangwill (2020) understands epistemic pluralism concerning knowledge as the claim that very different facts may constitute knowledge. In this paper, I argue primarily for pluralism about *epistemic values* by arguing that knowledge is not of the highest or even of outstanding epistemic value.^3^ Moreover, I favor a form of pluralism concerning epistemic concepts and epistemic phenomena according to which not only traditional epistemic concepts such as knowledge, truth, and justification should be the subject of epistemic investigations, but also rather neglected epistemic concepts and phenomena. In this paper, I do not directly argue for this form of pluralism, but this view is indirectly supported by rejecting one potential argument against epistemic pluralism, namely that knowledge should be the sole focus of epistemic inquiry in virtue of having outstanding value when compared with other epistemic standings.

The project of investigating epistemic phenomena other than knowledge is neither incompatible with a more traditional epistemology that aims at defining knowledge in other epistemic terms such as belief, truth and justification, nor with knowledge-first epistemology as championed by Williamson (2000).^4^ However, it has an impact on the relative importance of these alternative epistemological projects. The less relevant the concept of knowledge is compared to other epistemic concepts, the less relevant these projects are in epistemology.

Kvanvig is one proponent of the view that there is too strong a focus on the concept of knowledge in contemporary epistemology. He explains this focus via the importance of skepticism in epistemology. Kvanvig (2003, 186) diagnoses that “skepticism provides the impetus for the origin of the discipline and has dominated the efforts of epistemologists throughout the history of epistemology, either in defense of skepticism or by way of rebuttal. The result of this dominance is that the discipline of epistemology has focused on the concept of knowledge and whatever constituents it has, such as truth and justification.” Kvanvig thinks that the increasing importance of commonsense philosophy in the tradition of Thomas Reid and G.E. Moore in epistemology tends to lead to agreement that the skeptical position is false and allows “a freedom of exploration in epistemology, unencumbered by any need to address constantly the arguments of the skeptics” (Kvanvig, 2003, 187).^5^ Thus, if epistemologists are not continually caught up in answering skeptical worries about knowledge, then they will have more resources to dedicate to other epistemic phenomena.

In this paper, I will contribute to this project of epistemic pluralism by focusing on the value of epistemic states. The value problem in epistemology, also called the Meno problem, focuses on the value of knowledge which can lead to a neglect of other epistemic standings. These analyses of the value of knowledge neither explicitly nor implicitly entail that knowledge is the epistemic stance with the highest value. Klein (2012 and 2014) argues that knowledge is “the most highly prized form of true belief” but many value theorists about knowledge do not explicitly endorse this claim. Nevertheless, it has to be noted that mainstream epistemic value theory widely ignores the idea that there might be epistemic standings that have a higher value than knowledge. I will argue that in terms of value knowledge does not have any outstanding value among other epistemic concepts, such as checking or proving in court, which are rather neglected. In Sect. 2, I will outline the value problem of knowledge and variants of it. In Sect. 3, I will discuss the values of checking, persuading and transferring knowledge, and proving in court. I will argue that these values often exceed the value of knowledge and are independent of it. I will defend this view in Sect. 4 against potential objections.

## The Value Problem of Knowledge

The specific attention paid to knowledge within epistemology is manifested in discussions of epistemic value, which focus to a large extent on value problems for knowledge. Different versions of this problem can be distinguished. The original Meno problem is the problem of why knowledge is more valuable than mere true belief. Pritchard (2007) calls this the primary value problem of knowledge and distinguishes it from the secondary value problem of knowledge, the question of why knowledge is more valuable than any proper subset of its parts. Moreover, Pritchard, Turri, and Carter (2018) also identify a tertiary value problem for knowledge, the question of why knowledge is *qualitatively better* than any epistemic standing falling short of knowledge.^6^ Each of these problems addresses the relationship between the value of knowledge and the values of its parts.

The epistemic mainstream discussion about epistemic value focuses on the value of knowledge and on the question of how this value is related to the values of its parts. It also discusses, often in passing, the value of truth, belief, and justification, which are usually assumed to be constituents of knowledge. There is wide agreement in epistemology that true belief has a certain value, although there is a discussion about whether truth and belief themselves are of value.^7^ Appealing to a new evil demon argument, Madison (2017) argues that justification can be of epistemic value that is not merely instrumental to truth, concluding that justification is valuable for its own sake. Hence, we can say that the value discussion in epistemology, focuses (1) on the value of knowledge, (2) on the value of the constitutive parts of knowledge such as belief, truth, and justification and (3) on the relationship between the value of knowledge and the value of its constitutive parts.^8^ These discussions mainly center on the question whether the value of knowledge exceeds the values of its parts or is equivalent to them. Hence, the mainstream value discussions in epistemology focus on the value of knowledge and on the values of epistemic states which are expected to be lower than, or at most equal to, the value of knowledge. In these discussions, there are not taken to be any epistemic standings whose values might exceed the value of knowledge.

Kvanvig (2003) chooses an alternative path in reflecting on the value problem in epistemology. He answers the question raised by the Meno problem by thoroughly arguing that the value of knowledge does not exceed the value of its parts. He emphasizes that this diagnosis does not entail that knowledge does not have any value at all. In contrast, knowledge is valuable, but its value simply does not exceed the values of its constitutive parts. Kvanvig argues that it is understanding, not knowing, that is of distinctive value. He maintains that knowing does not entail understanding since there can be knowing without the more global, coherentist features required for understanding. Furthermore, understanding does not entail knowing since understanding can involve elements of epistemic luck whereas knowing cannot. Hence, according to Kvanvig, knowledge does not imply understanding and vice versa. Understanding has a genuine, irreducible value whereas knowledge does not have any value that is independent of the value of its parts.

I share Kvanvig’s diagnosis that knowledge plays too large a role in contemporary epistemology and that other epistemic phenomena that are interesting in their own right are neglected without good reason. In this paper, I will argue that knowledge does not have any distinctive value compared to other epistemic phenomena and states. I will not contest the claim that knowledge has a genuine value that is higher than the value of any sum of its parts. In this respect, I will not contribute to the various versions of the value problem of knowledge in this paper. However, I will present and discuss further epistemic states or standings that are valuable and I will argue that their values exceed the value of knowledge. The states that I will discuss are checking, persuading and transferring knowledge, and proving in court.^9^ These cases are not meant to be exhaustive. Presumably, there may be other epistemic states whose values exceed the value of knowledge.

In this paper, I will argue against the claim that the value of knowledge is outstanding when compared with the value of other epistemic standings. Let’s say that the value of knowledge is outstanding iff at least one of the following two conditions is fulfilled:

(C1) Knowledge has the highest value of all epistemic standings.

(C2) Knowledge has some primary value in that the value of all other epistemic standings is derived from the value of knowledge.

One might accept C1, that there are epistemic standings of higher value than knowledge, but object with C2, that their value is derived from knowledge in that they consist in knowledge plus additional intellectual work. In order to argue against C2, I assume that C2 is false if there are epistemic standings concerning *p* (1) that do not entail knowing that *p* and (2) even if one does not know that *p* the value of the epistemic standing exceeds the (potential) value of knowing that *p*. I assume that this conjunction is a sufficient condition for the falsity of C2, but I leave open whether it is also necessary. Hereinafter, I will argue against C1 and C2. Moreover, since I will argue for a pluralism about epistemic value, I will also argue against the claim that understanding is of outstanding epistemic value by arguing that it is neither of highest nor of primary epistemic value.

In this paper, I argue primarily against the claim that the value of knowledge is outstanding when compared with the value of other epistemic standings. I argue derivatively for epistemic pluralism in the sense that epistemology should not only focus on concepts such as knowledge, but also explore rather neglected phenomena such as checking, proving, understanding, explaining, arguing, convincing, or discriminating. For achieving this second point, it suffices to argue that these epistemic phenomena also have value. However, an advocate of epistemic monism could still object that the focus of orthodox epistemology on knowledge is justified because knowledge has outstanding value in the above sense. In order to meet this objection, and to make a stronger case for epistemic pluralism, it is necessary to argue that epistemic standings such as checking or legal proving can be of higher value that is independent of knowing.

One obvious consequence of this investigation is that knowledge is not, as Klein (2012 and 2014) claims, the most highly prized form of true belief. Moreover, even if knowledge has genuine value that is higher than the value of each proper subset of its constituents, in terms of values, knowledge does not play a crucial role. In some contexts, some other epistemic phenomena are more highly prized. Thus, in terms of value, it is not clear why knowledge should play such a crucial role in our epistemic life as most epistemologists think it does.

Kvanvig argues against the centrality of knowledge by contending that knowledge does not have any genuine value but that understanding does. On the one hand, I will extend this project by analyzing the value of further epistemic states, in particular checking, persuading and transferring knowledge, and proving in court. On the other hand, I will depart from this project by leaving open whether knowledge has genuine value. I think it is not necessary to take a stance on the value problem of knowledge for arguing that, in terms of value, knowledge does not have a special status on the epistemic landscapes. Rather it is sufficient to show that there are epistemic phenomena whose values knowledge exceeds but that there also phenomena whose values exceed that of knowledge.^10^

The discussion about epistemic *goals* is related to the discussion about epistemic values. A central goal is holding true beliefs (and not holding false beliefs.) Here, it is a crucial question whether other epistemic goals, such as holding justified beliefs or knowing, are only instrumental with regard to the goal of holding true beliefs or whether these are independent final goals.^11^ This discussion about epistemic goals slightly diverges from the discussion about epistemic values. For example, one can accept that holding true beliefs is the only final epistemic goal but nevertheless accept that knowledge has a higher epistemic value than mere true belief because it guarantees a more stable connection between belief and truth. Hence, pluralism about epistemic values is consistent with monism about epistemic goals.^12^ In this paper, I will leave open whether holding true beliefs is the primary or even the only epistemic goal. Checking, transferring knowledge and proving in court are all related to truth, but this holds of almost all epistemic standings. I will thus argue for pluralism about epistemic *values* while leaving open whether monism about epistemic *goals* is true.

I will also remain neutral on whether knowledge is of final or merely of instrumental value. However, I will argue that, if knowledge is of final value, then the other epistemic standings discussed here are also of final value, and, if knowledge is only of instrumental value, then so are these other epistemic standings. In either case, knowledge is not of outstanding epistemic value.^13^

## The Value of Other Epistemic Standings

In this section, I will focus on C1 and discuss epistemic standings which are neglected in epistemology in comparison to knowledge but whose values plausibly exceed the value of knowledge. These epistemic standings are, checking, transferring knowledge, and proving in court. In the following section, I will refute two objections that can be raised against these cases, thereby also rejecting C2.

### Checking

Let me first present a case where we value that a subject has checked that *p* was true more than we value mere knowledge that *p*.

**PILOTA (CHECKING)**

Pilota is pilot. As a pilot, she is committed to checking all engines of her plane before takeoff. Pilota knows that the engines worked perfectly during all the previous flights. Due to her background knowledge and her life-long experience, she knows that engine problems do not arise out of the blue. Thus, Pilota has inductive knowledge that the engines of her aircraft work properly. Nevertheless, she is obliged to check whether the engines work properly before takeoff, and we value this checking higher than we value her inductive knowledge that the engines work properly..

Before undergoing the checking procedure, Pilota, ex hypothesi, did not check that the engines work properly. However, based on her background knowledge, she plausibly has inductive knowledge that the engines still work properly at this point. This outcome is intuitively correct since it can be the case that S knows that *p* without having checked that *p* is true or that S can be in a position to know that *p* without being in a position to check whether *p* is true. In order to make this point, I am only committed to accepting this plausible claim about the relation between knowing and checking and that there is inductive knowledge.

However, this result fits well with a systematic theory of checking, developed in Melchior (2019), which can explain in more detail why Pilota knows that the engines work properly even though she did not check. I define successfully checking as follows:

S *checked that p* was true via M *iff*

1. S intentionally used M for determining whether *p* is true.
2. M is a checking method with respect to *p*.
3. M accurately indicates that *p*.^14^

I argue that M is a checking method with respect to *p* only if M is sensitive with respect to *p*, i.e. if, in the nearest possible worlds where M is used to determine whether *p* is true and where *p* is false, M does not indicate that *p*. This sensitivity condition was introduced by Nozick (1981), who argues that sensitivity together with the further modal condition of adherence are necessary and sufficient for converting a true belief into knowledge.

Nozick’s claim that sensitivity is a necessary condition on knowledge has been heavily criticized. Vogel (1987) and Sosa (1999) argue that induction typically yields insensitive beliefs even though we have inductive knowledge.^15^ They present cases of inductive knowledge that is insensitive as counterexamples against Nozick’s claim that sensitivity is necessary for knowing and conclude that sensitivity accounts of knowledge, which commit us to rejecting inductive knowledge in paradigmatic cases, must be rejected. I argue in Melchior (2019) that sensitivity is necessary for checking but not for knowing and that, therefore, sensitivity marks a crucial distinction between knowing and checking. Notably, PILOTA is a paradigmatic example of inductive knowledge, which is presented by critics against sensitivity accounts of knowledge.^16^ Thus, those authors who accept that the subjects know in the cases presented by Sosa and Vogel should also accept that Pilota knows that the engines work properly. According to this picture, Pilota did not check via induction that her engines work properly, but she has inductive knowledge that they work properly. What we intuitively want in aviation is not only inductive knowledge about the reliability of airplanes based on experiences. We want a higher standard to be fulfilled, which is met in cases of careful checking procedures. Therefore, checking is not only of value in addition to knowing, its value intuitively exceeds the value of knowing.^17^ Accordingly, we have an intuitive case where it is more valuable to have checked that *p* than to know that *p*.

### Transferring Knowledge

Transfer of knowledge is a second example of an epistemic standing which is, in certain contexts, of higher value than knowledge. We can persuade others in the sense that we make others form beliefs, for example by demonstrating and/or arguing for a particular proposition. Beliefs resulting from persuasion can be true or false, justified or unjustified, and they can constitute knowledge or fail to do so. Persuading somebody is a particular action which can be of value. For example, one can persuade a climate change denier about the existence of climate change and thereby make her change her actions. However, not every act of persuasion obviously has value. If A persuades B via demonstration and/or argumentation that *p* and *p* is false, then this action might not have any genuinely valuable effects and might not count as an achievement of A. A might even persuade B with the aim of deceiving B about a false proposition. In this case, A’s persuasion manifests a vice rather than a virtue at least prima facie. Suppose now that A persuades B about a true proposition *p*, i.e. A makes B believe a true proposition. If true beliefs have a particular value, then so does causing a true belief in someone else. B might still lack knowledge that *p* in this case, e.g. because A does not have any good evidence for *p* and believes that *p* via mere guessing. Importantly, persuasion can also create knowledge, namely testimonial knowledge. Take the following case:

**Testimonial knowledge**.

A persuades B that *p* via demonstration and/or argumentation and B thereby acquires knowledge that *p*.

If knowledge is of particular value, then making someone else know is also valuable.

One dispute about testimonial knowledge centers on the question of whether knowledge can be generated by testimony or only be transmitted. Preservationists assume that testimonial knowledge is always transferred knowledge, i.e. B can acquire testimonial knowledge that *p* via testimony from A only if A also knows that *p*. Non-preservationists like Lackey (2008) object that one can also acquire testimonial knowledge from someone who does not know the target proposition and that therefore testimonial knowledge is a genuine form of knowledge.^18^ If one takes a preservationist stance, then making someone else know implies that one also knows and the value of making someone else know plausibly exceeds the value of knowing. In case of non-preservationism, this entailment does not hold. In this case, we can distinguish between A making B a knower, which does not imply knowledge of A, from transfer of knowledge:

**Transferring knowledge**

A transfers knowledge that *p* to B iff A knows that *p* and A persuades B that *p* via demonstration and/or argumentation and B thereby acquires knowledge that *p*..

A transfers knowledge that *p* to B only if A knows that *p*. If knowing that *p* has a specific value, then the value of transferring knowledge *prima facie* exceeds this value. I only claim that this is prima facie the case. There might be specific circumstances where the transfer of knowledge is not more valuable than merely knowing. For example, transferring knowledge of how to build a nuclear weapon among terrorists is plausibly not valuable. However, in the absence of such circumstances, it seems highly plausible that transferring and thereby extending knowledge is of additional value compared to mere knowledge. Hence, we can find epistemic standings in the field of testimony whose values arguably exceed the value of mere knowing.

### Proof in Court

Let me next come to a third type of epistemic standing whose value plausibly exceeds the value of knowledge, cases of proving, in court. Take the following case:

**LAWLA (PROVING in COURT)**

Lawla is an attorney who is defending X. There is strong evidence presented in court that X is guilty of murdering Y. However, Lawla can find a piece of evidence that clearly shows that X cannot be the murderer. Unfortunately, the evidence is inadmissible, since Lawla obtained it illegally, e.g., from an illegal website or by breaking into someone’s house and stealing it. Consequently, the evidence is dismissed in court. X is sentenced to life in prison. Lawla and everybody else who is informed about the dismissed evidence know that X did not commit the crime. However, Lawla could not prove that X is not guilty..

In this example, Lawla knows that X did not commit the crime. What we value, however, is whether she could prove it. In particular, it would be highly valuable if Lawla were able to prove that X is not guilty. Proving that X is not guilty in court is more valuable than Lawla’s mere knowledge that X is not guilty.^19^

One line of argumentation against the claim that legal proof can be of higher value than knowledge and that its value is not derived from the value of knowledge is to argue that knowledge simply *is* the aim or norm of legal proof.^20^ In this case, it is hard to see why the value of legal proof should exceed the value of knowledge or be independent of it. This knowledge view of legal proof, however, has been criticized in a number of ways. Enoch et al. (2021) regard it as knowledge fetishism and defend a non-epistemic and incentive-based version of legal proof and Enoch et al. (2021) contest more generally the epistemic project of legal epistemology.^21^ Another way of avoiding the problem of collapsing legal proof into knowledge is by stressing that knowledge is factive whereas legal proof is not.^22^

I think these are valuable objections against the view that knowledge is the norm of legal proof, but for the purposes of this paper, we can make a different point. In the example presented, Lawla fails to provide a legal proof due to evidence that is inadmissible for purely juridical reasons. These juridical reasons need not be epistemically relevant in any significant respect, and it would be highly implausible to claim that inadmissible evidence generally precludes one from knowledge. For example, there are no reasons to assume that evidence that has been acquired illegally cannot yield knowledge. Notably, defenders of the knowledge view of legal proof such as Moss (2022) do not reflect on inadmissible evidence. I can hardly believe that they would commit themselves to the view that one cannot know via inadmissible evidence. Hence, inadmissible evidence provides a clear criterion for separating knowledge from legal proof.

Let me recap at this point: I argue for the claim that the value of knowledge is not outstanding among the value of other epistemic standings. I assume that it is outstanding if (C1) knowledge has the highest value of all epistemic standings or (C2) the value of all other epistemic standings is fundamentally based on the value of knowledge. We have identified various candidates for epistemic standings that are plausibly more valuable than mere knowing, namely checking, transferring knowledge, and proving in court.

In this paper, I remain neutral about some central questions about epistemic value. I do not defend a particular view on why knowledge is valuable and do not take a stance on whether epistemic standings are of final or only of instrumental value. For establishing my point, it is sufficient to show that, if knowledge is valuable according to a specific theory about epistemic value, then so are these other epistemic standings. Analogously, I only need to show that, if knowledge is of final value, then these other epistemic standings are of higher final value and, if knowledge is only of instrumental value, then these other epistemic standings are of higher instrumental value.

One popular theory about epistemic value is virtue theoretic. According to this view, knowledge is a form of achievement and achievements are always finally valuable. Perhaps it is disputable whether knowledge is an achievement, since knowledge is not obviously an action that manifests a competence, but checking, transferring knowledge and proving in court undoubtedly involve actions and are, consequently, undoubtedly achievements. Hence, if one opts for a virtue theoretic take on the value of knowledge, then checking, transferring knowledge, and proving in court are also valuable. Moreover, in each of the cases presented, the subject can know without having checked, having transferred knowledge, or having proven in court. The values of the achievements of having checked, having transferred knowledge, and having proven in court plausibly exceed the value of knowing. Moreover, if knowledge derives its value not from being an achievement but from being connected to virtuous character traits such as diligence, integrity, or love of truth, as Zagzebski (2003) champions, then plausibly also checking, transferring knowledge, and proof in court derive their values from these connections. Hence, if one accepts a virtue theory about the value of knowledge, either action-based or character-based, then one must also ascribe value to checking, transferring knowledge, and proving in court. And, given the examples presented, one is also committed to accept that these values exceed the value of knowledge. Admittedly, there are further theories about the value of knowledge on the market, but plausibly any of these theories should regard checking, transfer of knowledge, and proofing in court in the cases presented as more valuable.

Let me come back to issues concerning instrumental value and final value. One might argue, against virtue theoretic accounts, that actions like checking the engines of an aircraft are only instrumentally valuable with regard to the final value of saving lives. Analogously, proof in court is only instrumentally valuable with regard to the final value of doing justice. I do not find this line of argumentation entirely implausible but, importantly, I do not see why knowledge should be of final value in these cases if checking and proving in court are only of instrumental value. Hence, if epistemic standings such as checking or proving in court only have instrumental value, then plausibly knowledge only has instrumental value.

In this paper, I focus on arguing against the view that knowledge is of outstanding value. Kvanvig (2003) shares this view, but in virtue of arguing for the claim that understanding, not knowledge, is the epistemic standing that is of crucial value. As previously mentioned, I defend pluralism about epistemic values in that I think there is not a single epistemic standing that is of crucial value, neither knowing nor understanding. The values of checking, transferring knowledge, and proving in court can exceed the value of understanding as they can exceed the value of knowing. For example, it is more valuable to check that the engines of an aircraft work properly than merely to understand why they work properly; it is more valuable in certain contexts to transfer knowledge than only to understand the known proposition and finally it is more valuable to provide a proof in court that someone is innocent than merely to understand that she is. Thus, the acquired results for the value of knowledge also hold mutatis mutandis for understanding.^23^

## Two Objections

In this section, I address two objections that can be raised against the claim that the epistemic standings presented are more valuable than mere knowing. The first one is anti-intellectualist, i.e., contextualist or subject-sensitivist in nature; the second has it that the value of the other epistemic standings is derivatively based on the value of knowledge such that knowledge is the epistemic standing of distinctive value. The first objection can be used in defense of C1, that other epistemic standings are not of higher value than knowledge. Via the second objection, one can try to reestablish C2, that knowledge has some primary value such that the value of all other epistemic standings is derived from the value of knowledge.

### The anti-intellectualist Objection

One potential objection against the cases presented here is anti-intellectualist in spirit. There is a contextualist and a subject-sensitive invariantist version of this objection. Contextualism is the view that in different ascriber contexts different standards for knowledge are in play. Ascriber contexts are specified by the practical interests of the knowledge ascriber. In ordinary or low-stakes contexts, ordinary standards for knowledge hold, but when the stakes go up, as in high-stakes cases, the standards for knowledge also go up.^24^ Subject sensitive invariantism or SSI, as defended by Hawthorne (2004), Stanley (2005), and Fantl and McGrath (2009), agrees with contextualism that knowledge is not only determined by evidence but also by practical interests. However, they disagree with contextualism about whose practical interests matter. Contextualism claims that the practical interests of the knowledge ascriber matter whereas SSI holds that the practical interests of the believers matter.^25^ Stanley (2005) calls the traditionalist epistemological picture that only evidence but not practical interests matter intellectualism while Fantl and McGrath (2009) call it purism. I follow Stanley’s terminology. According to this terminology, contextualism and SSI are two branches of anti-intellectualism.

Here is an obvious objection against the analysis of PILOTA from an anti-intellectualist perspective. PILOTA is a paradigmatic high-stakes case (according to contextualism and SSI) since the lives of numerous people are at stake with every flight. Therefore, the standards for knowing are extraordinarily high in these contexts. In fact, the level for knowledge is raised up to a level such that Pilota’s background knowledge plus induction is not sufficient for knowing that the engines work properly. Hence, it is false according to contextualism and SSI that Pilota knows. Having checked that the engines work properly is necessary for knowing in this high-stakes context. However, what we value in the case of PILOTA is knowledge and not that someone has checked, but in high-stakes cases knowledge requires having checked.^26^ I remain neutral here about whether contextualism, SSI, or intellectualism provide the correct analysis of the verb ‘knows’, although I think there are good reasons for drawing a conceptual distinction between knowing and checking.^27^ Thus, I am not convinced by this contextualist objection against the analysis provided about PILOTA, nevertheless it is a possible move. Importantly, we will see that this contextualist objection is not equally applicable in other cases.

Let’s have a look first at knowledge transfer. Transfer of knowledge that *p* requires that some audience believes that *p*. A speaker A can persuade a hearer B about the truth of *p* in various different ways.^28^ For example, A can simply assert that *p* and B believes that *p* because B trusts A. Moreover, A can persuade B by arguing or demonstrating that *p* is true. However, persuasive argumentation or demonstration requires that A and B share a common ground prior to engaging in argumentation. This common ground plausibly involves agreement about the premises, agreement about the reliability of sources used, and agreement about the validity or cogency of the arguments presented. Without such a common ground, A will fail to persuade B.^29^

Importantly, A can also fail to persuade B that *p* even if A is in a perfect epistemic position concerning *p*, i.e., if A knows all the premises of her arguments, knows that the arguments presented are rational, knows that the sources used are reliable, and so on. B, for example, might be paranoid and distrust A no matter which argument or demonstration A provides. However, it is highly implausible in this case to claim that A does not know that *p* only because A cannot persuade B. Knowledge and the capacity of transferring knowledge easily come apart.^30^ Accordingly, it is implausible to assume that there are contexts where ‘A knows that *p*’ is true only if A can transfer this knowledge to B by making B believe that *p* via argumentation. This holds for attributor-contexts and contextualism as well as for believer-contexts and SSI. Hence, the anti-intellectualist objection, which might be raised in the case of checking, is not cogent for knowledge transfer. Analogously, it is highly implausible to claim that Lawla does not know that X is not guilty simply because the conclusive evidence was inadmissible for purely juridical reasons. Hence, the anti-intellectualist objection might be plausible in the case of checking, but it is clearly not convincing in cases of transferring knowledge and in cases of proving.

### The Derivation Objection

I suggest that knowledge is of outstanding value among epistemic standings iff its value exceeds the value of other epistemic standings (C1) or the values of other epistemic standings are based on it (C2). So far, I have mainly focused on rejecting the first condition. Let me now reflect on the second. A subject S transfers knowledge if she knows that *p* and makes another subject know that *p*. If knowing that *p* is of a particular value, then plausibly knowing that *p and* making someone else know that *p* is of higher value.

Transfer of knowledge is valuable at least partly because knowledge itself is valuable. If knowledge were not valuable at all, then transferring knowledge would plausibly also lack value. Accordingly, we have to admit that transferring knowledge exceeds the value of mere knowing but that knowing could be of outstanding value in that the value of transferring knowledge is based on the value of knowledge. This line of objection is reasonable for knowledge transfer, but importantly it cannot be generalized to the other epistemic standings discussed. In particular, the value of proving that X is not guilty is not derivatively based on the value of knowing that X is not guilty. Suppose that Lawla is a lawyer who is mistakenly convinced that X is guilty although X happens to be innocent. Nevertheless, because of her professional attitude, Lawla manages to correctly prove that X is not guilty. In this case, Lawla does not believe that X is not guilty and therefore fails to know, as Stella in Lackey’s (2008) case of the creationist teacher. However, her legal achievement is valuable although Lawla does not know. Hence, the value of proving in court is not based on the value of knowing.

A similar point can be made for checking. Suppose that Pilota is a highly reliable pilot who strictly follows the required procedure of checking whether the plane works properly before every take off. Moreover, after running through the checking procedure, Pilota would also correctly report that she checked that the plane works properly. Suppose further that Pilota is also very anxious and fails to actually believe what the checking procedure indicates, namely that the plane works properly. Since Pilota fails to believe, she also fails to know. Nevertheless, we value the fact that she checked and this value is not derived from any knowledge.

In this paper, I argue against the claims that knowledge has the highest value of all epistemic standings (C1) and that knowledge has some primary value in that the value of all other epistemic standings is derived from the value of knowledge (C2). I assume that C2 is false if there are epistemic standings concerning *p* (1) that do not entail knowing that *p* and (2) even if one does not know that *p* the value of the epistemic standing exceeds the (potential) value of knowing that *p*. We have seen that the first condition is fulfilled, since there can be instances of proving in court and checking without knowing, due to lack of belief. Moreover, the value of Lawla’s proof in court and the value of Pilota’s checking are not influenced by the fact that both fail to know. Thus, (2) is also fulfilled because the successful legal proof as well as the successful checking procedure exceed the potential value of knowledge also in those cases where knowledge is missing due to lack of believing.

I have introduced two potential objections against the view that there are epistemic standings such as checking, transferring knowledge and proving in court, which are of higher value than mere knowing. The first, anti-intellectualist, objection has it that we always value knowledge but that the standards for knowledge can vary with the context (either of the knowledge ascriber or the believer) such that in some contexts knowledge requires attaining these other epistemic standings. We have seen that this objection might be plausible in the case of checking. I leave open whether intellectualism or anti-intellectualism about knowledge is true, but if one opts for anti-intellectualism, then one might argue that Pilota is in a high-stakes case such that she knows that the engines work properly only if she has checked. Consequently, we do not value checking higher than knowledge in this case because knowing requires having checked in this context. However, we have also seen that this anti-intellectualist objection is not convincing for knowledge transfer or proof in court. Whether we make someone know that *p* not only depends on our epistemic position but also on whether the hearer eventually believes the target proposition. If the persuader and the hearer do not share enough common ground, then plausibly the hearer will not believe the target proposition even if the persuader is knowledge-wise in a perfect epistemic proposition concerning the target proposition. Hence, there is no plausible context where knowing that *p* requires transfer of knowledge. The same holds in Lawla’s case. It is implausible to assume that Lawla does not know that X is not guilty just because some evidence was dismissed in court.

The second objection concerns the dependence of the value of epistemic standings on the value of knowledge. This is arguably the case for knowledge transfer, which is valuable partly because knowing itself is valuable. However, this is not the case for proof in court, whose value is not based on knowledge being valuable. Hence, the anti-intellectualist objection might be convincing for checking, but not for transferring knowledge or for proving in court. The derivation objection has some plausibility for transferring knowledge (and also for checking given that one opts for anti-intellectualism), but not for proof in court.

It might be subject of argumentation whether C1 or C2 holds for some of the cases discussed. However, none of the two conditions C1 and C2 for knowledge being of outstanding value is fulfilled for all epistemic standings. Crucially, there are also epistemic standings such as proof in court for which undoubtedly neither C1 nor C2 holds. These epistemic standings are of higher epistemic value than mere knowledge and they are of independent value, regardless which particular view on knowledge and its value one takes; and presumably other epistemic standings, which neither fall under C1 nor under C2 can be found.

## Conclusion

It is a widely held view that knowledge has value. Different value problems, can be distinguished -- why knowledge is more valuable than mere true belief or why it is more valuable than any proper subset of its parts. These discussions focus on the value of knowledge and its constitutive parts. These approaches suggest, at least implicitly, that the value of knowledge is distinctive compared to the values of other epistemic standings in that knowledge is the highest prized epistemic standing, or at least outstanding in the sense that other epistemic standings are value-wise based on it. I have argued that this view is mistaken. Neither is knowledge the epistemic standing with the highest value, nor are the values of other more highly-prized epistemic standings based on its value. In this sense, knowledge is not of any distinctive value in the field of various epistemic standings. Reflections on values do not support the view that knowledge has a special status among the large variety of epistemic standings. Either knowledge does not have distinctive status among other epistemic standings or we must find an explanation for its distinctive position elsewhere.

## Footnotes

1 See Klein (1981) and Lehrer (1990) for accounts of defining knowledge in terms of undefeated justification.

2 There is an increasing interest in epistemology in theories of understanding. See Kvanvig (2003). For a theory of checking, see Melchior (2019), for a theory of discrimination, see Melchior (2021).

3 As I will argue in the following section, this pluralism about epistemic values does not entail a pluralism about epistemic goals or goods.

4 For a collection of discussions on knowledge-first epistemology, see Carter, Gordon, and Jarvis (2017). For a critical discussion of knowledge-first epistemology, see McGlynn (2014).

5 I find Kvanvig’s explanation of the focus on knowledge in epistemology plausible and share his view on the importance of opening up epistemology to explorations of concepts other than knowledge and its constituents. However, I need not accept his explanation of why we focused so far on knowledge in epistemology for the purposes of this paper.

6 The terminology surrounding the Meno problem is not always entirely clear. Kvanvig (2003, xiii) calls the Meno problem “the problem of how, and whether, knowledge has a value that exceeds that of its parts.” This formulation comes closer to the secondary value problem than to the primary one.

7 The view that both truth and belief have a certain value is defended by Kvanvig (2003).

8 There are some exceptions, for example Kvanvig (2003).

9 I understand in this paper epistemic states or epistemic standings in a rather wide sense, that also includes actions such as checking, proving, and transferring knowledge and the status of having successfully performed these actions. One might object that it is misleading to compare knowledge to checking, proving, and transferring knowledge since knowledge is a state whereas the other epistemic standings happen to be events. I do not find this objection convincing because having checked, having proven or having transferred knowledge are state-like and they are the result of an achievement as is knowledge.

10 One option for arguing that knowledge does not have any distinctive value compared to other epistemic standings is simply to argue that knowledge and other epistemic standings do not have any particular value, either because epistemic standings are not valuable or because, more generally, value nihilism is true and there is no value at all. In this paper, I want to argue for the claim that, in terms of value, knowledge does not have any distinctive standing in the epistemic landscape. Hence, if one opts for any such nihilist view about epistemic values or about value more generally, then the aim of this paper is trivially achieved. In the following, I will ignore these nihilist views. However, the following line of argumentation can be understood as conditional: If knowledge has value at all, then it does not have any distinctive value in the landscape of epistemic standings.

11 David (2005) defends the view that true beliefs are the only epistemic goal, while Kvanvig (2005) and DePaul (2001) object to this view.

12 See Bondy (2020).

13 There are different accounts on the market of why knowledge is valuable. Virtue epistemologists defend the idea that there is an immediate connection between knowledge and achievement. Greco (2003 and 2008) maintains that knowledge is creditable true belief. Sosa (2007) develops a general framework for actions and achievement and holds that achievements are finally valuable. It is S’s achievement to hold a true belief iff the true belief is aptly formed. Thereby, virtue theorists can formulate a straightforward account of the value of knowledge within a broader framework of actions and achievement. For a criticism of this virtue-theoretic account, see Piller (2012). Zagzebski (2003) champions another strand of virtue epistemology. She argues that the value of knowledge can be explained by not only focusing on true belief but also by taking virtuous motives for acquiring true beliefs into account, such as diligence, integrity, or love of truth. In this paper, I will not take any stance on why knowledge is valuable. Consequently, I will not criticize or defend any view concerning the source of the value of knowledge. However, I will show that, *if* a certain account specifies knowledge as valuable, then it also specifies the other epistemic standings discussed as valuable as well.

14 Having checked *that p* was true is meant to be an abbreviation for having checked whether *p* was true with the result that *p* is true.

15 This view has been recently challenged by Wallbridge (2018). However, Wallbridge focuses on Sosa’s (1999) thrash chute case. His discussion does not affect PILOTA. For a more general discussion of sensitivity and induction, see Melchior (forthcoming a).

16 For further examples of inductive beliefs that plausibly constitute knowledge, see Vogel (1999 and 2012).

17 One potential reason for the higher value of checking is that it fulfills sensitivity whereas knowing need not meet this condition. Also Pilota’s checking method is plausibly sensitive, i.e., the method would not indicate that certain engines function properly if they were not to function properly. I will not argue here in detail for the assumption that only sensitive methods are adequate for checking. For a defense of this claim, see Melchior (2019). For the purposes of this paper, it is sufficient to rely on the clear intuition that inductive knowledge does not suffice in contexts like aviation and that we value undergoing a distinct checking procedure.

18 Lackey (2008) presents the case of Stella, an undergraduate teacher. She presents the main theses of evolutionary theory to students who thereby acquire knowledge by testimony. However, Stella is a creationist who does not believe these main theses and consequently does not know them. Lackey concludes that testimonial knowledge does not require knowledge of the testifier. For objections against this view, see Faulkner (2011). For the terminology of preservationism and non-preservationism, see Green (2022).

19 One might claim that an analogous point can be made for proving in science by arguing that scientists often have knowledge that certain scientific hypotheses are true according to ordinary standards for knowledge even though the hypotheses are not proven according to scientific standards. In these cases, providing a proof for a scientific hypothesis is of additional value, which is often honored by prestigious scientific awards. Even though this is an interesting point, I am unsure whether this analogy is helpful. The independence of the value of proof in court from the value of knowledge depends on juridically inadmissible evidence that can, nevertheless, yield knowledge. I do not see any analogous procedural restrictions for scientific proving. Thus, it does not seem implausible to claim that scientific proofs aim at knowledge and that we have scientific knowledge of a hypothesis iff it is finally proven.

20 Versions of this view are defended by Blome-Tillmann (2017) and Moss (2022).

21 For recent criticisms of knowledge-based theories of legal proof, see Fratantonio (2021) and Mortini (2022). For a defense of this view and for criticisms of incentive-based accounts, see Gardiner (2019) and Moss (2022).

22 This view is defended by Blome-Tillmann (2015) and Smith (2018).

23 This diagnosis is meant to be compatible with the view that there are also contexts where the value of understanding exceeds in a certain way the value of checking, transferring knowledge, and proving in court.

24 For influential versions of contextualism, see Cohen (1988), DeRose (2009 and 2017), and Lewis (1996).

25 Obviously, these two views collapse in case of self-ascriptions of knowledge.

26 Pilota’s evidence based on background knowledge and induction is insensitive, but her method of checking the engines is plausibly sensitive. Combining a sensitivity theory with contextualism supports the view that, in ordinary contexts we can know based on insensitive evidence, but in high-stakes cases the standards for knowledge are moved up to a level such that a true belief must be sensitive for yielding knowledge. For a sensitivity-based version of contextualism, see DeRose (1995 and 2017). For a critical discussion of DeRose’s version of contextualism, see Melchior (2014 and 2019).

27 For a discussion of how knowing and checking relate to contextualism, SSI, and intellectualism, see Melchior (2019).

28 Transfer of knowledge can also happen if B already truly believes that *p* but comes to know that *p* by acquiring evidence from A that is necessary for converting B’s true belief into knowledge. These cases are not relevant for our purposes here.

29 A might present a supporting argument for the truth of the premises, the reliability of the sources, or the validity or cogency of the arguments presented, but, without a common ground, A inevitably enters an infinite regress and fails to persuade B. Hence, persuasive argumentation or demonstration requires this kind of common ground.

30 I do not see how one can convincingly argue in this case that in some contexts A fails to know that *p*, because A cannot provide a persuasive argumentation or demonstration for *p*. For a more detailed analysis of why knowledge and persuasive argumentation can come apart, see Melchior (forthcoming b).
